# Acid-Responsive Self-Healing Waterborne Epoxy Coating: Preparation, Release Behavior, and Anticorrosion Performance Based on Bowl-Shaped Mesoporous Polydopamine Nanocontainer Loaded with 2-MBI Inhibitors

**DOI:** 10.3390/polym17162265

**Published:** 2025-08-21

**Authors:** Xiaohong Ji, Minghui Yang, Huiwen Tian, Jin Hou, Jingqiang Su, Zhen Wang, Zixue Zhang, Yuefeng Tian, Liangliang Zhou, Guanghua Hu, Yunfei Yang, Jizhou Duan, Baorong Hou

**Affiliations:** 1 State Key Laboratory of Advanced Marine Materials, Key Laboratory of Marine Environmental Corrosion and Bio-Fouling, Institute of Oceanology, Chinese Academy of Sciences, Qingdao 266071, China; 2 Key Laboratory of Marine Chemistry Theory and Technology, Ministry of Education, College of Chemistry and Chemical Engineering, Ocean University of China, Qingdao 266100, China; 3 Shandong Railway Investment Holding Group Co., Ltd., Jinan 250014, China; 4 Jiqing High Speed Railway Co., Ltd., Jinan 250014, China; 5 Lunan High Speed Railway Co., Ltd., Jinan 250101, China

**Keywords:** acid-responsive coating, self-healing, bowl-like mesoporous polydopamine, corrosion protection

## Abstract

We present a straightforward emulsion-induced interfacial anisotropic assembly method for in- situ synthesis of bowl-shaped, self-encapsulated mesoporous polydopamine (BMPDA) nanocontainers (M-M@P) loaded with 2-mercaptobenzimidazole (2-MBI). Results showed that the loading capacity of the bowl-shaped mesoporous polydopamine reaches 24 wt.%. The M-M@P exhibits a cumulative MBI release of 91.61% after immersion in a 3.5 wt.% NaCl solution at pH = 2 for 24 h, accompanied by a corrosion inhibition efficiency of 95.54%. Additionally, the acid-responsive M-M@P not only enables controlled release of MBI but also synergistically promotes the formation of a protective film on the carbon steel substrate via the chelation of PDA-Fe^3+^, thereby enhancing the self-healing performance of waterborne epoxy coatings.

## 1. Introduction

Metal corrosion has emerged as a significant factor influencing the sustainable development of national economies and societies. Particularly, the corrosion of metals in acidic environments has garnered considerable attention globally [1]. In marine environments, the penetration of chloride ions can induce localized corrosion such as pitting and crevice corrosion. These processes can significantly lower the local pH through autocatalysis [2,3]. Moreover, oxidation is a common corrosion process in acidic environments. The oxidation reactions occur when metals are exposed to oxygen and water, resulting in oxide formation and the release of hydrogen ions. These ions lower the pH of the solution, rendering it acidic [4]. In addition, the activities of some microorganisms may cause corrosion and create an acidic environment [5]. For example, microbial activity of sulfate-reducing bacteria can reduce sulfuric acid to sulfide, leading to the release of acidic substances and causing environmental acidification. The presence of sulfides through the discharge of acidic wastes and acid rains may also create an acidic environment [6].

Various strategies have been implemented to control metal corrosion in acidic environments [7]. Among them, coating the metal surface is considered one of the most effective, low-cost, and environmentally friendly methods to prevent the corrosion of metals in acidic electrolytes [8]. In particular, water-based epoxy coatings are a pivotal development area in the coatings industry. This is primarily attributed to their minimal content of volatile organic compounds (VOCs), low emission, and alignment with energy conservation and environmental protection [9]. Nevertheless, prolonged exposure of the cured coatings to corrosive environments can lead to the formation of micro-damage and cracks. These can facilitate the penetration of corrosive agents such as water and oxygen, thereby accelerating corrosion reactions between the metal substrate and these agents. This process ultimately results in material failure and degradation of the coating’s barrier properties [10,11,12]. Exploring smart coatings with active self-healing capabilities is an effective strategy to eliminate this defect.

To endow waterborne epoxy (WEP) coatings with self-healing capabilities and extend their service life, extensive research has focused on enhancing their protective performance under damaged conditions [13,14]. Rather than restoring the structural integrity of the coating itself, the self-healing function is intended to provide sustained corrosion protection to the damaged metal surface [15]. In addition, corrosion inhibitors have traditionally been employed to mitigate corrosion and enhance the longevity of metals [16]. However, the direct incorporation of corrosion inhibitors into polymer coatings is constrained by their adverse impact on curing reactions and coating integrity.

Corrosion inhibitors are the most widely studied self-healing additives for coatings. Usually, corrosion inhibitors are encapsulated in micro-nano containers before being incorporated into the coating [17]. These carriers allow for the controlled release the corrosion inhibitors [18]. The inhibitors are stored in nanocontainers to avoid direct interaction with the coating structure, thus preventing many limitations [19]. However, microcapsules typically range from tens to hundreds of micrometers in diameter, making them unsuitable for thin coating applications. Furthermore, the poor mechanical properties of microcapsules and their incompatibility with the polymer matrix can compromise the performance of the composite coatings. A promising approach to address this issue is to encapsulate the corrosion inhibitor in nanocontainers. Nanocontainers, such as mesoporous titanium dioxide [20], carbon nanotubes [21,22], zirconium oxide [23], mesoporous silica [24], and polydopamine [25], have been studied for loading corrosion inhibitors into them. He et al. [26] employed a simple strategy to modify zeolitic imidazolate framework 8 (ZIF-8) with conductive polypyrrole (PPy) using in situ polymerization. The synthesized PPy@ZIF-8 composites were added into epoxy resin (EP) as active fillers to prepare long-lasting anticorrosion coatings. The nanocontainers have a wider range of structures and properties. When corrosion inhibitors are efficiently loaded, the involved nanocontainers can respond to triggering factors such as pH [27,28], thermal responsiveness [29], and aqueous media [24]. When these internal/external stimuli are triggered, the corrosion inhibitors can be released from the nanocontainers. Additionally, incorporating a barrier within the coating is an approach to prevent the corrosion inhibitor from leaching out, i.e., allowing the controlled release and enhancing the durability and self-healing capabilities of the coating against corrosion. Bai et al. [30] demonstrated a new strategy to construct pH-responsive nanocontainers as gatekeepers, which consisted of a laminated double hydroxide (LDH) surface with in situ grown ZIF-8 containing 2-mercaptobenzimidazole (MBM) as a corrosion inhibitor. The core shell structure of the composite filler exhibited excellent barrier properties and pH-responsive controlled release in epoxy (EP) coatings. Since corrosion changes the pH value of the corroded site and this process, in turn, affects the corrosion reaction, pH is considered a key triggering factor [31].

The common challenges with nanocontainers are their low loading capacity and slow responsiveness. The approach to solve the above-mentioned challenges lies in designing and preparing nanocontainers to achieve high loading capacity and rapid stimulus-responsive release. Recently, dopamine has attracted increasing attention, inspired by mussel adhesion proteins [32]. Because of their chemical reactivity, biocompatibility, and low cytotoxicity, dopamine finds wide application across various fields, including drug delivery, catalysis, and energy [33,34,35]. Dopamine exhibits intrinsic pH responsiveness, and its functional groups hold significant potential for enhancing the adhesion of nanoparticles to flat surfaces [36]. The MPDA with a large volume of mesopores and specific surface area can enhance loading capacity and facilitate rapid release. The formation of polydopamine (PDA) coatings occurs in an alkaline solution without requiring external stimuli such as light or heat. The uniformity of these coatings is governed by the diffusion of PDA and the reactivity of the surface [25]. The presence of unreacted catechol groups, which persist following the oxidative polymerization of dopamine, results in a high density of hydroxyl groups on the surface of nanocontainers. This modification significantly improves the wettability of the nanocontainers, which is particularly important for those used in water-based coatings [25]. Additionally, catechol groups play a significant role in self-healing coatings. Studies have shown that damaged polymer networks can be repaired through catechol-Fe^3+^ coordination bonds [37]. Furthermore, research has highlighted that salt-mediated cation–π interactions are crucial in the adhesion mechanisms of mussels [38]. Polydopamine-based microcapsules (PMs) were synthesized by self-polymerizing dopamine (DA) onto the surface of oxidized SiO_2_ particles, followed by the removal of the template core [39]. Subsequently, benzotriazole (BTA), a well-known corrosion inhibitor, was encapsulated inside the PM capsules (PMB) through adhesive interactions between BTA and the catechol groups present in PDA. The findings demonstrated that the combination of BTA and PMs imparted both self-healing and anticorrosive properties to the coating structure. Liu et al. [40] designed and synthesized a smart corrosion inhibitor carrier 2-mercaptobenzimidazole-Zn^2+^-polydopamine@graphite (MZPG) with an excellent pH response. The MZPG/EP coating maintained good corrosion resistance under acidic conditions due to the pH-responsive release of the corrosion inhibitor.

**Figure 1:**
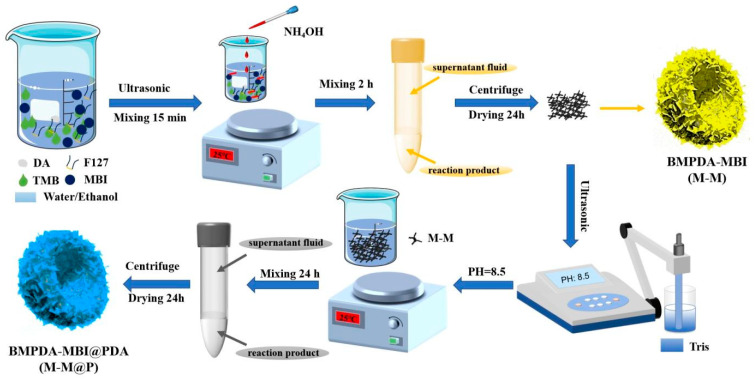
The synthesis mechanism of M-M@P.

This study focuses on employing the multifunctional emulsion-induced method to design the structure of mesoporous polydopamine, optimize its synthesis process, and regulate the fabrication of BMPDA nanocontainers. The objective is to achieve a high loading capacity of corrosion inhibitors by creating nanocontainers with large mesopores and significant specific surface area. Here, 2-MBI, a heterocyclic organic corrosion inhibitor, is loaded using a straightforward one-step method to optimize its incorporation process and enhance inhibitor loading efficiency. Self-coated nanoparticles are composed of BMPDA and PDA. Unlike other PDA-coated nanoparticles used for anticorrosion (e.g., PDA-coated mesoporous silica nanoparticles), both the core and the shell of M-M@P are composed of PDA. The self-encapsulation strategy simplifies the preparation method and avoids potential compatibility issues caused by complex systems. Coating the surface with a polydopamine layer prevents the loss of corrosion inhibitor from the nanocontainer into the coating. The M-M@P can accelerate inhibitor release in the acidic microenvironment, which may be due to the pH sensitivity of the PDA shell. PDA can act as a dispersant to ensure good interfacial compatibility between the nanocontainers and waterborne epoxy resin [41,42]. The synergy inhibition effect from PDA and MBI endows the coating with active protection properties, which effectively prevent the corrosion reactivity occurring at the coating–metal interface and improves the inhibition capability of coatings to micro corrosion pits. The synthesis mechanism and process of M-M@P are shown in Figure 1. The morphology, structure, loading capacity of MBI, sustained release, corrosion resistance, and self-healing characteristics of M-M@P have been examined comprehensively. The findings indicate that the combined effects of MBI and PDA endow the coatings with both self-healing and anticorrosive properties, thereby highlighting their potential for use in corrosion protection applications.

## 2. Experiments

### 2.1. Materials

Triblock polyethylene oxide-b-polypropylene oxide-b-polyethylene oxide (F127, Mw 12700) was obtained from Sigma-Aldrich (St. Louis, MO, USA). 1,3,5-Trimethylbenzene (TMB), dopamine hydrochloride(DA), tris(hydroxymethyl)aminomethane (Tris), ammonia (NH_4_OH), and 2-MBI were sourced from Shanghai Aladdin Biochemical Technology Co., Ltd. (Shanghai, China). All chemicals utilized were of laboratory grade with a purity exceeding 95%. Sodium chloride (AR) and ethanol (AR) were supplied from Sinopharm Chemical Reagent Co., Ltd. (Shanghai, China). The MU-618 waterborne epoxy resin (epoxy equivalent 200–220 g/mol) and CU-600 curing agent (amine value 180–190 mg KOH/g) were provided by Shanghai Rundan New Material Technology Co., Ltd. (Shanghai, China). The coatings were applied to pre-cleaned Q235 carbon steel substrates. Q235 carbon steel, used as the substrate in this study, has a chemical composition of C, 0.15; Mn, 0.06; Si, 0.35; S, 0.01; P, 0.55; Fe, addition (in wt.%).

### 2.2. Preparation of BMPDA

The BMPDA nanocontainers were synthesized using a multifunctional nano-emulsion assembly method. The detailed procedure is as follows. First, 1.0 g F127, 1.5 g DA, and 2 mL TMB were dispersed in a 100 mL mixture of deionized water-ethanol (DI water: ethanol mixing ratio = 1:1 *v*/*v*) under ultrasonic treatment for 2 min to form an emulsion. Then, 3.75 mL aqueous ammonia was dropped into the reaction mixture under stirring to promote the self-polymerization of DA. After stirring for 2 h, the BMPDA particles were collected by centrifugation, washed several times with water and ethanol, and then dried at 60 °C.

### 2.3. Loading of MBI

The BMPDA nanocontainers loaded with the corrosion inhibitor MBI were synthesized through a simple one-step in situ method. Specifically, 1.0 g F127, 1.5 g DA, and 2 mL TMB were dispersed in a 100 mL deionized water-ethanol mixture (DI water: ethanol mixing ratio = 1:1 *v*/*v*) under 2 min of ultrasonic treatment to achieve a homogeneous emulsion. Then, 1.8 g 2-MBI was added (initial F127/2-MBI unit molar ratio = 1:5). Subsequently, 3.75 mL of aqueous ammonia was slowly added under stirring. After the reaction mixture was stirred under strong magnetic stirring at 25 °C for 2 h, the BMPDA-MBI (M-M) was collected by centrifugation, washed three times with deionized water and ethanol, and finally dried at 60 °C. To form the protective polydopamine (PDA) layer, 0.4 g DA was completely dissolved in the M-M suspension, followed by the addition of 0.15 g Tris to promote further DA self-polymerization. The polydopamine (PDA) layer was obtained on the surface of the M-M nanoparticles. After stirring for 24 h, the BMPDA-MBI@PDA (M-M@P) nanoparticles were separated by centrifugation and washed with ethanol. The collected M-M@P nanoparticles were then subjected to ultrasonic treatment in ethanol for 30 min to remove the template. This template removal step was repeated three times. Finally, the purified M-M@P nanoparticles were dried at 60 °C.

### 2.4. Fabrication of Coatings

Q235 carbon steel substrates were sequentially abraded using 400, 800, and 1200 grit sandpapers and then subjected to ultrasonic cleaning in ethanol for 10 min to eliminate surface organic contaminants. The cleaned substrates were then rinsed with acetone and kept in a vacuum environment for later use. For the preparation of the epoxy resin mixture, 70 g of waterborne epoxy resin was first diluted with 10 mL of deionized water to lower its viscosity, forming a homogeneous solution. Subsequently, 0.07 g of M-M@P nanoparticles was added and dispersed by magnetic stirring at room temperature for 30 min, followed by ultrasonic treatment for 10 min to ensure uniformity. Next, 35 g of curing agent was incorporated. The mixture was stirred for 15 min, subjected to an additional 10 min of ultrasonic treatment, and then treated under vacuum for 10 min to enhance the dispersion of nanoparticles and to remove any air bubbles in the uncured composite. The prepared coating mixture was painted on pre-cleaned and air dried steel substrates using a paint brush. The coated samples were dried at room temperature (relative humidity 10–30%) for 7 days and then cured in an oven at 80 °C for 90 min to prepare WEP/M-M@P coating samples. For comparison, a blank epoxy coating without M-M@P was prepared and named pure WEP. The coating sample added with M-M was named WEP/M-M. The final thickness of the dried coating was about 60 ± 5 μm.

### 2.5. Characterization

To prepare samples for observation with a thermal field divergence scanning electron microscope (SEM, FEI Nova, nano 450, Tokyo, Japan), the dried nanoparticles were first dispersed in an ethanol solution at a concentration of 0.1 mg/mL. This dispersion was then applied to a clean silicon wafer. Prior to SEM observation, the samples were sputter-coated with a 20 nm layer of gold. The microstructure and chemical composition of the samples were analyzed using SEM with energy dispersive X-ray spectroscopy (EDS). The morphologies of BMPDA, M-M, and M-M@P nanoparticles were further examined using a high-resolution transmission electron microscope (TEM, JEOL JEM-2100PLUS, Peabody, MA, USA). Fourier transform infrared spectroscopy (FTIR, Nicolet iS10, Waltham, MA, USA) was employed to identify the components within the nanoparticles, with measurements conducted at a resolution of 1 cm^−1^, 64 scans, and across a spectral range of 4000–400 cm^−1^. The functional groups and structures of the synthesized BMPDA, M-M, and M-M@P nanoparticles were characterized using ultraviolet–visible spectroscopy (Hitachi U-3900H, Tokyo, Japan). Thermogravimetric analysis (TGA, Netzsch TG209F3, Selb, Germany) was carried out from 25 to 800 °C at a heating rate of 10 °C/min under a nitrogen flow of 100 mL/min. X-ray photoelectron spectroscopy (XPS, Thermo Fisher Escalab 250Xi, Waltham, MA, USA) measurements were performed with Al Kα radiation at 15 kV and 12 mA. The XPS spectra were calibrated against the C 1s peak at 284.6 eV to analyze the crystal and chemical structures of the samples. Additionally, the microstructure and chemical composition were examined using a high-angle annular dark field (HAADF) imaging system (Oxford, UK) equipped with an EDS detector. The release behavior of MBI was studied using UV–vis spectrophotometer (Hitachi U-3900H, Tokyo, Japan). Laser confocal microscopy (LSCM, CA, Lext OLS5000, Olympus, Waltham, MA, USA) and Raman spectrometer (Renishaw MZ20-FC, Gloucestershire, UK) were used to analyze the morphology and chemical composition of the coatings, respectively. All tests were conducted three times on three similar samples to ensure repeatability.

### 2.6. Release Behavior of Inhibitors from M-M@P Nanocontainers

To investigate the release characteristics of the encapsulated materials under different conditions, 10 mg of M-M@P nanoparticles were suspended in 40 mL of a 3.5 wt.% NaCl solution. These nanoparticles were subsequently enclosed in dialysis bags with a molecular weight cutoff of 1000 Da, and these bags were subsequently immersed in 100 mL of the same NaCl solution under continuous stirring. The pH of the release medium was adjusted to 2, 4, and 7 with the dropwise addition of HCl solution. Following different soaking periods, 3 mL samples of the dialysate were extracted for analysis using a UV–visible spectrophotometer. In this experiment, the characteristic absorption peaks of MBI were monitored to evaluate its release profile. For quantitative analysis, MBI standard solutions with concentrations of 0.002, 0.005, 0.010, and 0.020 mg/mL were prepared in 3.5 wt.% NaCl solution. The absorbance of each solution was measured at the maximum absorption wavelength of MBI. A standard calibration curve was constructed by plotting absorbance versus concentration, and linear regression was performed to obtain the corresponding standard equation. The release amount of MBI at different time points was determined by measuring the absorbance of the dialysate and applying the standard equation. This enabled the evaluation of MBI release behavior across different pH conditions. The expression is noted as follows:

(1) $$\begin{array}{c} Y\;=\;113.11\;X\;+\;0.0036 \end{array}$$

where *X* is the concentration (mg/mL), and *Y* is the UV absorbance.

### 2.7. Corrosion and Self-Healing Performance of WEP/M-M@P Coating

The assessment of the coatings’ anticorrosion properties was performed using electrochemical impedance spectroscopy (EIS) and potentiodynamic polarization curves. Electrochemical measurements were conducted using an Autolab PGSTAT302N workstation equipped with a standard three–electrode configuration. In this configuration, a platinum sheet (20 mm × 20 mm × 0.2 mm, surface area 400 mm^2^) was used as the auxiliary electrode, a saturated calomel electrode (SCE) serves as the reference electrode, and the coated steel samples function as the working electrode. The working electrode was embedded in epoxy resin, exposing a square area of 1 cm^2^. All electrochemical tests were started at room temperature (23 ± 2 °C). When conducting electrochemical tests, a shielding box was used to isolate external electromagnetic radiation. The shielding box was made of metal and has a good shielding effect. The inside of the shielding box was kept dry and clean to avoid additional interference to the electrochemical test. The process began with recording the open circuit potential (OCP) to ensure system stability [43]. Subsequently, EIS measurements were taken at the stable OCP over a frequency range of 10^−2^ to 10^5^ Hz, using an AC voltage amplitude of 5 mV. ZsimpWin 3.6 software was used to perform appropriate equivalent fitting of the experimental data. The inhibition efficiency based on the EIS measurements was calculated as follows [44]:

(2) $$\eta \;=\;\frac{R_{P}\;-\;R_{P}^{0}}{R_{P}}\;\times \;100\%\;$$

where $R_{p}$ and $R_{p}^{0}$ represent the total polarization resistance in the presence and absence of nanocontainers loaded with corrosion inhibitors, respectively.

Linear sweep voltammograms were obtained by altering the electrode potential between −200 mV and +200 mV relative to the open circuit potential (OCP) with a scan rate of 0.5 mV/s. To ensure the precision of the results, each experiment was conducted in triplicate. *E*_corr_, *i*_corr_, anodic Tafel slope (*β*_a_), and cathodic Tafel slope (*β*_c_) were acquired via Tafel extrapolation fitting. The calculation of inhibition efficiency was performed as described below [39]:

(3) $$\eta \;=\;\frac{i_{corr}^{0}\;-\;i_{corr}}{i_{corr}^{0}}$$

where $i_{\mathrm{corr}}$ and $i_{\mathrm{corr}}^{0}$ are the corrosion current densities of coated carbon steel with and without nanocontainers loaded corrosion inhibitors in the salt solution, respectively.

Electrochemical impedance spectroscopy (EIS) serves as a highly effective method for evaluating the self-healing properties of coatings. In this study, scratches with dimensions of 10 mm by 0.2 mm were created using a scalpel. Subsequently, these scratched coatings were immersed in 3.5 wt.% NaCl solutions across various pH levels to assess their self-healing performance. Prior to each EIS measurement, an open circuit potential (OCP) test was conducted for 30 min to ensure stability, and the perturbation voltage amplitude was increased to 10 mV to enhance the stability of the test data, due to the high impedance of the coating system. The total polarization resistance $R_{p}^{0}$ (the sum of the coating resistance *R_p_* and the charge transfer resistance *R_ct_*) is used to evaluate the corrosion protection performance and self-healing efficiency of the scratched coating. The calculation formula is shown in Equation (2). In a similar manner, electrochemical measurements were performed on three different samples to ensure reproducibility. The impedance spectra were processed and analyzed using ZSimpWin software. Furthermore, a scanning Kelvin probe (SKP) was employed to evaluate the potential difference between the sample and the probe in the area surrounding the scratches.

## 3. Results and Discussion

### 3.1. Characterization of BMPDA, M-M, and M-M@P

**Figure 2:**
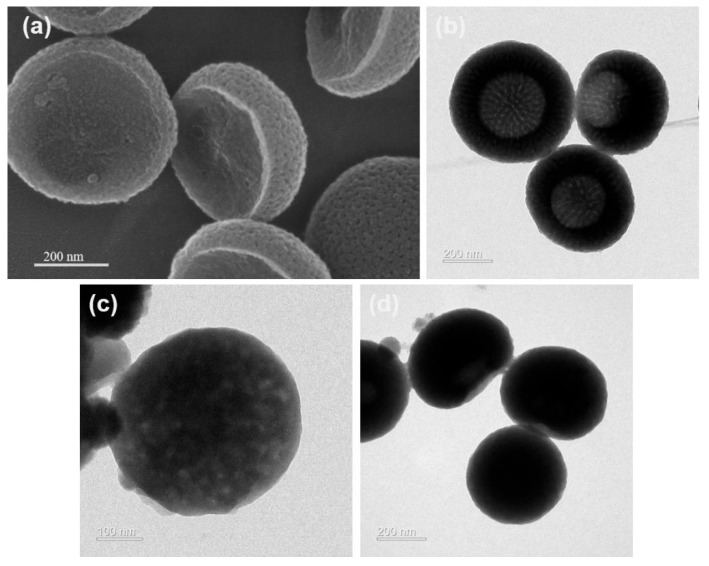
SEM image of (**a**) BMPDA; TEM image of (**b**) BMPDA, (**c**) M-M, and (**d**) M-M@P.

The morphology of the synthesized nanomaterials is shown in Figure 2. Figure 2a,b show that the BMPDA has a bowl-shaped morphology with a particle size of 250 ± 20 nm. The nanoparticles have a radially oriented and ordered large mesoporous structure and are arranged radially from the center to the surface. These mesopores are evenly distributed in the BMPDA nanoparticles to form a hollow multi-tubular structure with cylindrical open channels. This unique structure provides abundant space for loading corrosion inhibitors and offers enhanced responsiveness against the corrosive media. Figure 2c exhibits the morphology of the BMPDA after loading with MBI. It can be seen from the figure that the mesoporous structure of the particles before and after loading is significantly different. The shell of M-M shows a more uneven surface than the shell of BMPDA, which verifies the loading formation of MBI. It can be preliminarily judged that the MBI corrosion inhibitor is loaded in the mesopores of the BMPDA. The morphology of M-M@P is shown in Figure 2d, and an organic coating can also be observed on the edge of the BMPDA container. This PDA layer not only inhibits the migration of the corrosion inhibitor from the nanocontainer into the water-based epoxy resin but also imparts an effective pH-responsive capability to the nanocontainer.

**Figure 3:**
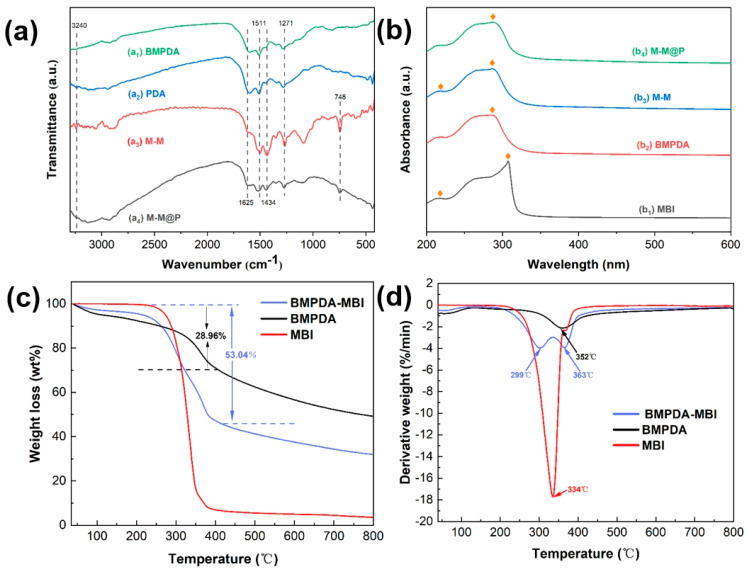
(**a**) FT-IR spectra of PDA, BMPDA, M-M, and M-M@P; (**b**) UV–vis absorption spectra of MBI, BMPDA, M-M, and M-M@P; (**c**) TG curves of MBI, BMPDA, and BMPDA-MBI; (**d**) DTG curves of MBI, BMPDA, and BMPDA-MBI.

FTIR spectra were employed to investigate the chemical composition of M-M and M-M@P. The FTIR spectrum of the PDA is shown in curve (a_1_) in Figure 3a, which has an absorption peak at 3240 cm^−1^ assigned to the stretching vibration of ortho-hydroxyl group of the benzene ring. The formation of intramolecular hydrogen bonds leads to this broad absorption peak. The peak around 1511 cm^−1^ results from the stretching vibration of the carbon–carbon double bond of the benzene ring skeleton. Curve a_2_ shows that the absorption peak of BMPDA is similar to that of PDA, indicating that the formation of mesopores may not change the chemical structure of PDA. The FTIR spectrum of M-M, shown in curve a_3_, exhibits distinctive absorption peaks corresponding to MBI and BMPDA. The peak at 1434 cm^−1^ is associated with the stretching vibrations of the C-H bonds in the benzene ring, while the peak at 748 cm^−1^ corresponds to the out-of-plane bending vibrations of hydrogen atoms in the benzene ring of MBI. These observations confirm that MBI has been successfully incorporated into the BMPDA [45]. The absorption bands at 1625 and 1511 cm^−1^ are related to the aromatic skeleton vibrations of the carbon–carbon double bond and the CN=N bond in the indole ring, respectively. The peak of dopamine was observed at 1271 cm^−1^ due to the formation of primary amine and C-O stretching vibration, proving successful self-polymerization of dopamine on the M-M surface [46].

The UV–vis spectra were recorded to examine the effective loading of MBI and the encapsulation by PDA (Figure 3b). In curve (b_1_), characteristic absorption peaks at 218 nm and 307 nm correspond to the presence of MBI [47]. Overall, the broad absorbance band of BMPDA, lacking distinct peaks, suggests that the structure and bonding in BMPDA are comparable to those found in naturally occurring melanin [48]. However, the curve (b_2_) of the BMPDA appeared around 283 nm, showing eumelanin-like characteristics and indicating successful polymerization of DA [49]. After loading MBI, the curve (b_3_) of M-M shows the characteristic absorption peaks of both PDA and MBI, pointing out the effective loading of MBI. In addition, the absorbance peak of M-M@P in curve (b_4_) only at 287 nm indicates that the DA has successfully self-polymerized on the surface of the M-M, indicating PDA successful encapsulation.

The TG analysis (Figure 3c,d) was performed to examine the loading efficiency of the prepared nanoparticles. As shown in Figure 3c, the initial weight loss of BMPDA below 100 °C is attributed to the evaporation of adsorbed water, likely due to the presence of hydrophilic groups in BMPDA [50]. After loading with MBI, the amount of adsorbed water decreases, which may result from the partial masking or interaction of MBI with the hydrophilic groups, thereby reducing their exposure to moisture [51]. In the temperature range of 100 °C~400 °C, M-M decomposes rapidly and is accompanied by a rapid mass loss. As depicted in the differential thermogravimetric analysis curve in Figure 3d, the decomposition peaks are observed between 299 °C and 363 °C, accompanied by a weight loss of approximately 53.04%. This significant decomposition primarily corresponds to the breakdown of MBI within the BMPDA container during this temperature range. The larger decomposition peak at 299 °C is caused by the superposition of MBI decomposition. Similarly, the weight loss of M-M fillers at 363 °C is related to the decomposition of OH- in BMPDA. The loading content of M-M was approximately 24.08%. The large mesoporous structure of the BMPDA and its significant specific surface area enhance its loading capacity for corrosion inhibitors. This confirms that BMPDA serves as an ideal reservoir for corrosion inhibitors. According to the TG and DTG analysis, BMPDA is inferred to demonstrate high efficiency in loading MBI.

**Figure 4:**
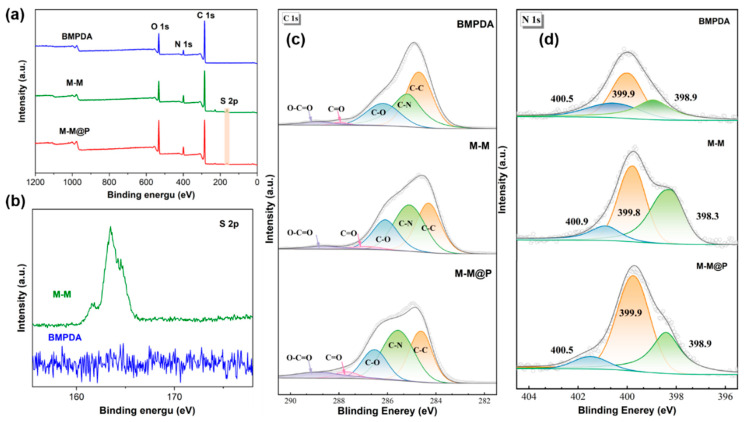
(**a**) BMPDA, M-M, and M-M@P XPS survey scans; (**b**) S 2p high-resolution XPS spectra of BMPDA and M-M; (**c**) C 1s high-resolution XPS spectra of BMPDA, M-M, and M-M@P; and (**d**) N 1s high resolution XPS spectra of BMPDA, M-M, and M-M@P.

XPS characterization was performed to further confirm the successful loading of MBI onto the BMPDA nanocontainers and the encapsulation effect of the PDA. The full XPS spectra of BMPDA, M-M, and M-M@P are shown in Figure 4a. All three samples exhibit characteristic peaks corresponding to C 1 s, N 1 s, and O 1 s. Notably, a distinct sulfur signal appears in the M-M spectrum, which is absent in pristine BMPDA, confirming the successful incorporation of sulfur-containing MBI molecules. In addition, the relative intensity of the N 1s peak has increased, indicating that the N content has increased; meanwhile, the oxygen content decreased after MBI loading. Upon encapsulation with PDA (M-M@P), the O 1s signal shows a significant increase in intensity, which can be attributed to the presence of hydroxyl and benzoquinone groups introduced by the self-polymerized dopamine structure.

Meanwhile, the intensity of the S 2p peak decreases, indicating that the outer PDA layer could effectively encapsulate the M-M.

To gain a more detailed understanding of the chemical structure of the synthesized nanoparticles, high-resolution spectra for C 1s and N 1s were analyzed. As shown in Figure 4c, it can be observed from the C 1s high-resolution spectrum that the C 1s peak can be fitted to five distinct peaks corresponding to C-C (284.6 eV), C-N (285.1 eV), C-O (286.1 eV), C=O (287.9 eV), and O-C=O (288.9 eV) bonds [52]. After loading with MBI, the M-M sample exhibits a notable increase in the C-N peak area, indicating the successful introduction of C-N bonds from MBI molecules. Furthermore, a further increase in the C-N signal is observed in the M-M@P sample, which can be attributed to the polydopamine (PDA) encapsulation process. This enhancement suggests that PDA was effectively grafted onto the M-M surface and that MBI was successfully incorporated. As shown in Figure 4d, it can be observed from the N 1s high-resolution spectrum that the characteristic deconvoluted peaks appear at 398.9, 399.9, and 400.5 eV, belonging to tertiary amine/aromatic hydrocarbon (-N=), C-N bond (containing amine group), and -NH_3_^+^/primary amine (-NH_2_), respectively [53,54]. The areas of the C-N and C=N peaks in the case of the M-M sample increase significantly due to the presence of nitrogen-containing MBI, providing further evidence of successful inhibitor loading. The C-N and -NH_2_ structures corresponding to the M-M@P increase further, while the area related to the deconvoluted peak of C=N decreases, indicating that the PDA has been successfully grafted onto the surface of the BMPDA-MBI.

**Figure 5:**
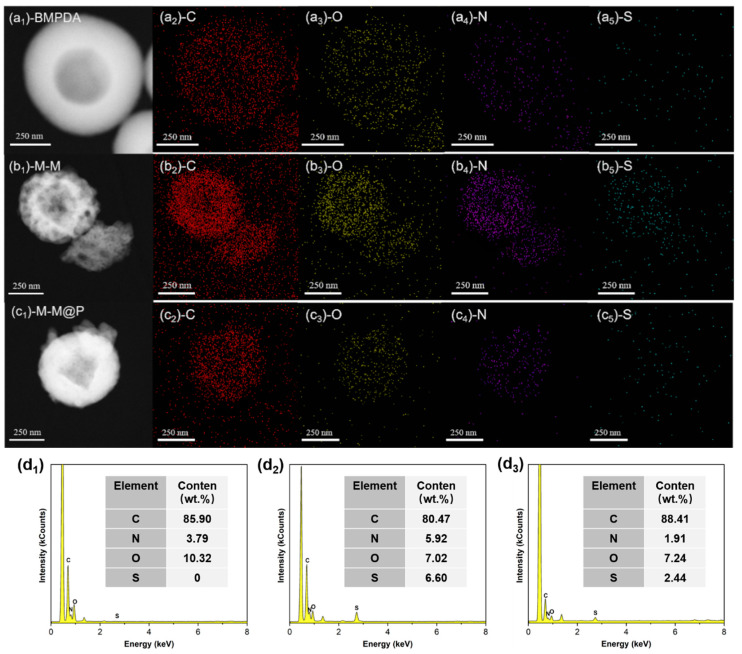
HAADF image of (**a_1_**–**a_5_**) BMPDA, (**b_1_**–**b_5_**) M-M, and (**c_1_**–**c_5_**) M-M@P; (**d_1_**–**d_3_**) elemental composition: (**d_1_**) BMPDA, (**d_2_**) M-M, and (**d_3_**) M-M@P.

The high-angle annular dark field scanning (HAADF) image and elemental mapping image of BMPDA, as shown in Figure 5a_1_–a_5_, clearly show its porous structure. The elements of C, N, and O are evenly distributed on the surface of BMPDA nanoparticles. As shown in Figure 5b_1_–b_5_, the elemental mapping of M-M shows that the elements of C, N, O, and S are uniformly distributed on the nanocontainer. The elemental mapping confirms the porous structure of BMPDA and the successful loading of the MBI as a corrosion inhibitor containing the characteristic sulfur element. In addition, the uniform distribution of S element confirms that the MBI is loaded into the mesoporous channels of the BMPDA. The HAADF image and the element mapping image of M-M@P are shown in Figure 5c_1_–c_5_. Compared to M-M, the S element signal in the M-M@P is significantly reduced or shielded, indicating that the PDA has successful encapsulated the M-M. As shown in Figure 5d_2_, the elemental composition of M-M is C (80.47 wt.%), N (5.92 wt.%), O (7.02 wt.%), and S (6.60 wt.%) according to the EDS results. The presence of S confirms the successful incorporation of the inhibitor (Figure 5d_1_), while the relative N content also increases, attributable to nitrogen-rich MBI molecules. Concurrently, the O content decreases, which is likely due to the partial replacement of oxygen-containing functional groups by MBI. However, the M-M@P (Figure 5d_3_) is affected by the hydroxyl and benzoquinone structures in dopamine due to the modification of PDA grafting, and the O element increases more noticeably while the S element decreases relatively. This is consistent with the XPS results.

### 3.2. Release Performance of M-M@P

When corrosion occurs on the metal surface, the local pH value of the surrounding environment will change. When the pH value changes, the pH responsive nanocontainer surface changes accordingly, thereby influencing the release of the corrosion inhibitor encapsulated within their structure [55]. To assess the release dynamics of MBI from the nanocontainers, the release behavior of M-M@P at various pH levels was examined using UV–vis spectroscopy. Specifically, the release performance of MBI from M-M@P samples was tracked by evaluating the absorbance intensity of the released MBI over time as a function of pH.

**Figure 6:**
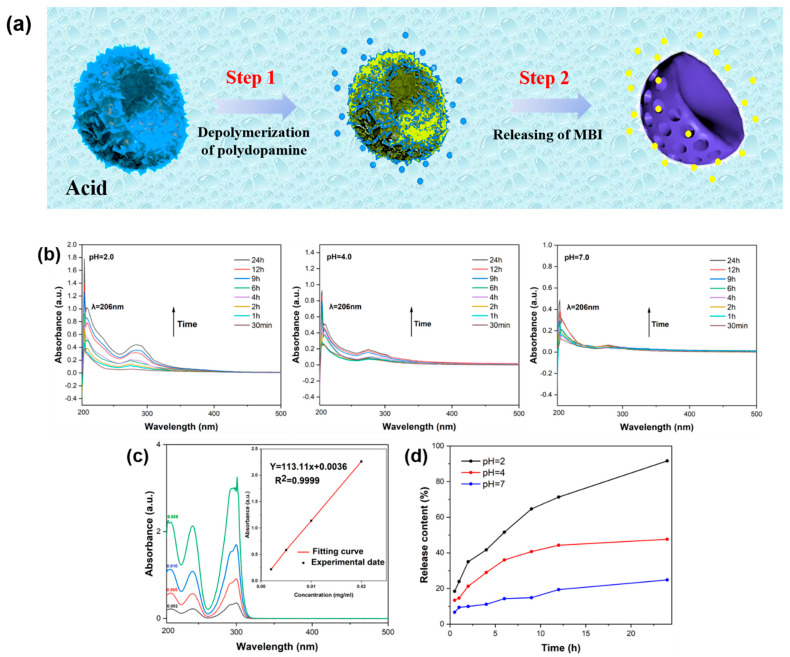
(**a**) Stimulus and response characteristics of M-M@P nanoparticles, (**b**) UV–vis absorption spectra of M-M@P in the 3.5 wt.% NaCl solution at different pH values, (**c**) UV–vis absorption spectra of MBI in the 3.5 wt.% NaCl solution and UV–vis absorption standard curves of pure MBI obtained by linear fitting (illustration), and (**d**) 24 h MBI release at different pH values.

As depicted in Figure 6a, the rate at which MBI molecules diffuse into a 3.5 wt.% NaCl solution was significantly enhanced under acidic conditions. The UV absorption intensity of the characteristic MBI peak at 206 nm served as a metric to assess the amount of MBI released from the nanocontainers. Using the established standard curve, a linear relationship between the UV absorbance at 206 nm and MBI concentration was determined (Figure 6c). This relationship enabled accurate calculation of MBI release amounts at different immersion periods (Figure 6d). Figure 6b illustrates that the M-M@P nanoparticles, when immersed in a 3.5 wt.% NaCl solution with varying pH, showed a gradual increase in peak intensity over time. Although the overall release trends were similar, the extent of release varied significantly with pH. Notably, acidic environments promoted higher release rates and intensities throughout the study period. After 24 h of immersion, the cumulative release rates of MBI at pH 7, 4, and 2 were 24.83%, 47.62%, and 91.61%, respectively. The initial release is primarily attributed to MBI molecules encapsulated within the inner mesoporous core of the BMPDA nanocontainers.

When pH = 7, the solubility of MBI is relatively low. As the pH decreases below 7, the solubility of MBI increases correspondingly. Under mildly acidic conditions (pH = 4), the release rate of MBI remains limited, with approximately 75% of the MBI molecules retained within the BMPDA nanocontainers. In contrast, in the external environment with pH = 2, the release rate of MBI is significantly accelerated within the first 24 h, showing a markedly enhanced release trend. After 12 h, more than 90% of MBI is released from the nanocontainer at pH = 2. The release behavior of MBI is closely related to the pH of the surrounding environment. On the one hand, the solubility of MBI increases as the pH decreases, which facilitates faster diffusion of MBI into the external solution [56]. On the other hand, the protonation of the amino group on the outer layer of PDA is destroyed under acidic conditions, resulting in the rapid release of MBI [25,57]. These observations confirm the successful encapsulation of MBI within BMPDA nanocontainers and clearly demonstrate their pH-responsive, stimuli-triggered release behavior.

### 3.3. Anticorrosion and Self-Healing Performance

#### Nanocomposite Coatings in NaCl Solution at Different pH Values

**Figure 7:**
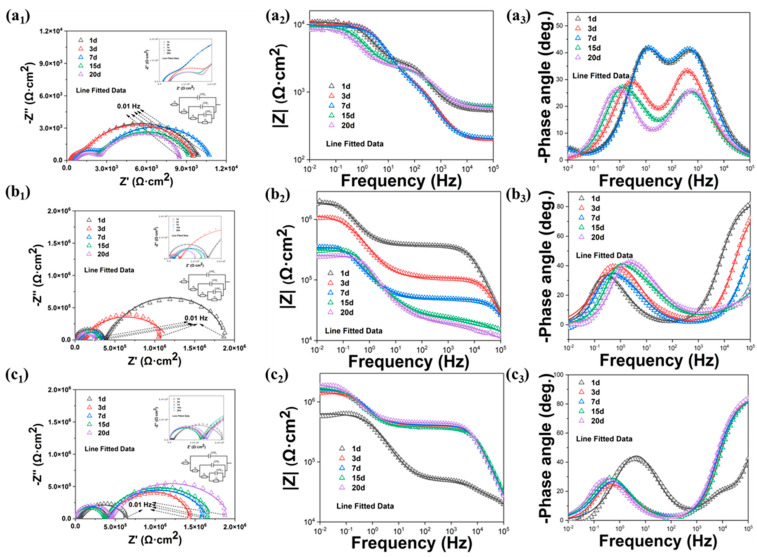
Nyquist and Bode plots of carbon steel electrodes coated with different coatings immersed in the 3.5 wt.% NaCl solution at pH = 2 after different times: (**a_1_**–**a_3_**) WEP, (**b_1_**–**b_3_**) WEP/M-M, and (**c_1_**–**c_3_**) WEP/M-M@P.

To investigate the self-healing properties of the coatings, artificial defects were introduced to accelerate corrosion. Figure 7 presents the EIS results of scratched WEP, WEP/M-M, and WEP/M-M@P during immersion in the 3.5 wt.% NaCl solutions at pH = 2. The corresponding EIS data for samples immersed at pH = 4 and pH = 7 are provided in Supplementary Figures S1 and S2. Generally, a higher impedance modulus at the lowest frequency indicates superior corrosion resistance and effective barrier properties.

Figure 7a_2_ illustrates that the |Z| value of the WEP coating at a frequency of 0.01 Hz decreases over time, from 1.06 × 10^4^ Ω·cm^2^ initially to 8.27 × 10^3^ Ω·cm^2^ after 20 days of immersion. This reduction indicates an accelerated deterioration of the carbon steel substrate and a rapid degradation of the WEP coating. In contrast, Figure 7b_2_ shows that the |Z| value for the WEP/M-M coating at the same frequency drops to 2.39 × 10^5^ Ω·cm^2^ after 20 days. Notably, as depicted in Figure 7c_2_, the impedance of the WEP/M-M@P coating increased on the third day. After 20 days of immersion, the |Z| value for the WEP/M-M@P coating was 1.84 × 10^6^ Ω·cm^2^, which is three orders of magnitude higher compared to the WEP coating. This higher impedance is primarily attributed to the PDA adhesion, which improves compatibility with waterborne epoxy resin, combined with the synergistic corrosion inhibition provided by MBI and PDA.

The phase angle in the high-frequency region is an important reference indicator for the corrosion resistance of the coating and the metal substrate [58,59]. Comparing Figure 7a_3_–c_3_, it is evident that the phase angle of the coating with nanofillers exceeds 80° in the high-frequency region. In contrast, the high-frequency phase angle of WEP remains very low, consistently below 10°. This discrepancy indicates that the WEP sample fails as immersion time increases. In particular, in Figure 7a_3_,b_3_, the phase angle in the high-frequency region also increased as the immersion duration increased. This observation further illustrates that the corrosion inhibitor within the nanocomposite coating exerted a repairing effect on the metal substrate. The repair film effectively hindered the penetration of corrosive media, demonstrating excellent anticorrosion and self-healing properties.

**Figure 8:**
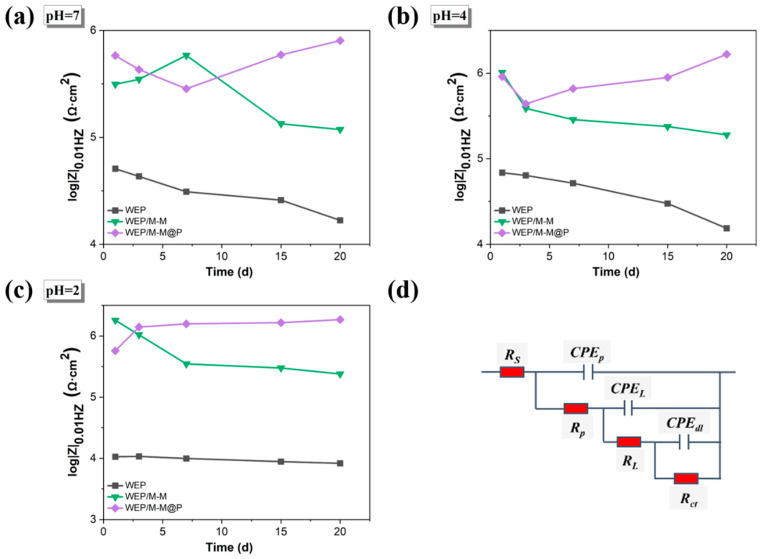
The |Z| value at a frequency of 0.01 Hz (|Z|_0.01Hz_) changes with time curve: (**a**) pH = 7, (**b**) pH = 4, (**c**) pH = 2, and (**d**) electrical equivalent circuit used for fitting EIS circuit.

All electrochemical results were fitted using the equivalent circuit in Figure 8d. In the fitted equivalent circuit, *R_S_*, *R_P_*, *R_L_*, and *R_ct_* represent the solution resistance, coating resistance, oxide layer resistance, and charge transfer resistance, respectively [60]. The parameters of *CPE_dl_* and *R_ct_* represent the interface corrosion process of carbon steel/coating. The parameters of *CPE_L_* and *R_L_* can be used to illustrate the formation of oxide layers, which may include corrosion products and passive films. The polarization resistance $R_{p}^{0}$ (the sum of oxide layer resistance *R_L_* and charge transfer resistance *R_ct_*) is used herein to evaluate the corrosion self-healing performance of the coating. Generally, the coating samples with good anticorrosion performance have higher *R_ct_* values. According to Table 1, the *R_ct_* value of WEP gradually decreases with the extension of immersion time, indicating that the interface corrosion reaction is active. The highest *R_ct_* value in the WEP/M-M@P coating sample is attributed to the rapid release and deposition of MBI on the scratch area after immersion for 3 days, indicating its excellent self-healing function. It can be seen from Table 1 that the anticorrosion performance of the WEP/M-M@P coating is better than that of other coatings during the entire immersion time. The lowest *R_L_* value belongs to the pure waterborne epoxy coating, indicating that the oxide layer formed on the substrate surface is relatively weak. For the coating containing WEP/M-M@P, its *R_L_* value is higher than that of the pure waterborne epoxy coating. This indicates that the presence of M-M@P nanoparticles within the structure of waterborne epoxy coating can enhance both corrosion resistance and self-healing properties of the coating. The *R_L_* of the WEP coating showed a decreasing trend, while that of the WEP/M-M@P showed an increasing trend. The higher *R_L_* values in WEP/M-M@P compared to the WEP indicate suppression of the electrochemical reaction at the interface. This suppression is attributed to the effective formation of MBI and PDA passivation films resulting from the released corrosion inhibitors at the scratches.

Comparing Figure 7 and Figures S1 and S2, it can be clearly seen that the barrier performance of the coating in the neutral 3.5 wt.% NaCl solution against corrosive media is generally lower than that of the coating sample in the 3.5 wt.% acidic NaCl solution. Figure 8a–c also show the trend of the |Z| value at a frequency of 0.01 Hz (|Z|_0.01Hz_) for different nanocomposite coatings at a frequency at different pH values. The maximum |Z|_0.01Hz_ for the WEP/M-M@P coating sample at pH = 7 is less than 10^6^ Ω·cm^2^. However the WEP/M-M@P coating at pH = 4 and 2 conditions exhibits excellent self-healing performance, and the |Z|_0.01Hz_ of them reach 1.66 × 10^6^ and 1.88 × 10^6^ Ω·cm^2^, respectively. From the test results of the blank coating and the nanocomposite coating, it can be concluded that MBI and PDA play a major anticorrosion role in the coating structure. As the pH gradually decreases, the WEP/M-M@P coating efficiently and controllably releases the MBI corrosion inhibitor on the carbon steel surface in the 3.5 wt.% acidic NaCl solution with a pH = 2. The resulting repair film effectively shields against corrosion reactions. The |Z|_0.01Hz_ for the WEP/M-M@P coating rebounded on the third day in the NaCl solution with pH of 2, showing better anticorrosion and self-healing properties than other pH values. Due to the intelligent repair characteristics of the coating in the acidic media, it has potential application value in the field of corrosion protection.

**Figure 9:**
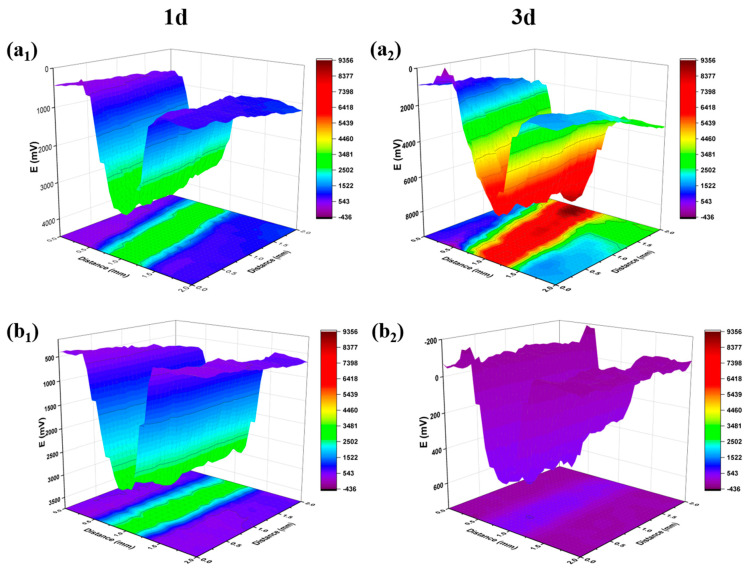
SKP images of (**a_1_**,**a_2_**) WEP/M-M@P and (**b_1_**,**b_2_**) pure WEP coating after immersion in 3.5 wt. % NaCl solution (pH = 2) for 1 d and 3 d.

To further verify the active corrosion protection ability of the coating, SKP tests were performed on (a) WEP/M-M@P coating and (b) WEP coating, both of which contained artificial scratches in 3.5 wt. % NaCl solution (pH = 2) in a rectangular area. SKP monitored the local surface condition of the sample by measuring the potential difference between the sample and the probe. SKP tests were performed on the scratched coating at 1 and 3 days, respectively. Figure 9 illustrates that a higher potential difference (ΔV) correlates with a decreased integrity of the coating. In Figure 9a_1_, after 1 day of immersion, the WEP/M-M@P-coated sample exhibited a maximum potential of 3177 mV and a minimum potential of 293 mV, resulting in a calculated ΔV of 2884 mV on the metal surface. Conversely, after 3 days of immersion (Figure 9a_2_), the maximum potential decreased to 642 mV, while the minimum potential dropped to −305 mV, giving a ΔV of 947 mV. In comparison, the WEP coating (Figure 9b_1_) showed a maximum potential of 3430 mV and a minimum of 84 mV after 1 day, resulting in a ΔV of 3346 mV. After 3 days, as shown in Figure 9b_2_, the maximum potential increased to 8920 mV, while the minimum potential decreased to −436 mV, yielding a ΔV of 9356 mV. The significant reduction in ΔV for WEP/M-M@P indicates a notable improvement in performance. This improvement is attributed to the corrosion in the scratched areas, which triggers the release of MBI and demonstrates the protective effect of the MBI corrosion inhibitor incorporated in M-M@P. The WEP/M-M@P coating thus exhibits significant self-healing capabilities.

**Figure 10:**
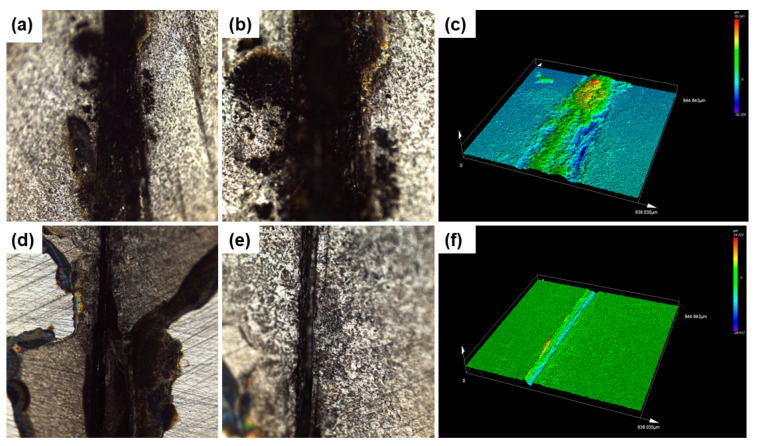
LSCM images from the surface of the coated specimens with artificial scratches after electrochemical testing (after peeling off the coating): (**a**–**c**) pure WEP-coated specimen and (**d**–**f**) WEP/M-M@P-coated specimen.

The LSCM technology is used to more intuitively analyze the self-healing performance of the coating, and the results are shown in Figure 10. Obvious corrosion, which appears as a large amount of yellow corrosion products, is observed in the scratch area of the pure WEP coating sample (Figure 10a–c), and the corrosion spreads around the scratch. The corrosion products are significantly higher than the scratch area as presented in the 3D morphology, indicating that the carbon steel in the scratch area is severely corroded. In contrast, the WEP/M-M@P coating specimens (Figure 10d–f) exhibit different surface characteristics. There is no obvious accumulation of corrosion products within or around the scratch area. In addition, the 3D morphology shows a fewer products in the scratch area, and the overall surface remains relatively clean and smooth. These observations indicate that the WEP/M-M@P coating has good self-healing properties and effectively inhibits the development and spread of corrosion.

**Figure 11:**
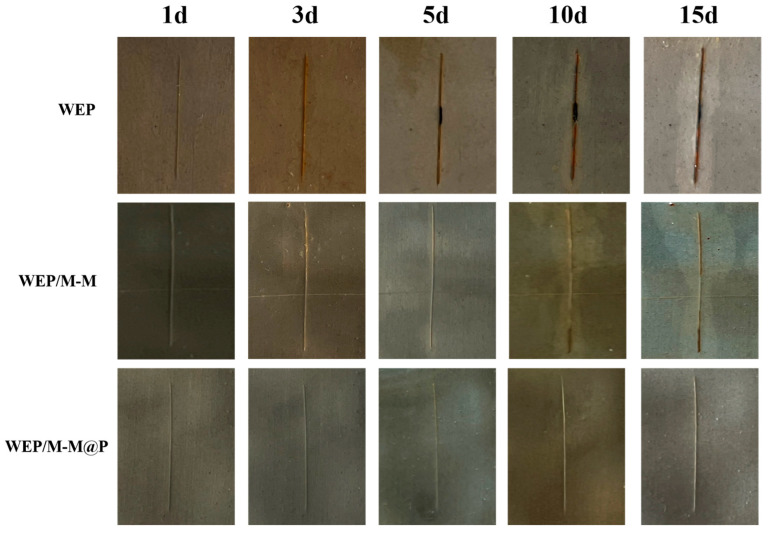
Optical images of pure WEP, WEP/M-M, and WEP/M-M@P coatings after immersion in the 3.5 wt.% NaCl solution at pH = 2 for 15 days.

The scratched specimens were directly immersed into a 3.5 wt.% NaCl solution with a pH of 2 to assess the corrosion resistance and self-healing capabilities of the coatings. Figure 11 illustrates that, after 3 days of immersion, significant corrosion products were observed in the crack regions of the WEP sample. As the immersion time increased, the protection ability of the WEP without nanofillers further deteriorated. The coating fell off from the steel substrate after immersion for 15 days due to the penetration of the corrosive medium, indicating that the adhesion strength of the WEP was weak. This outcome underscores the dual self-healing effect facilitated by MBI and PDA. However, visible corrosion products still developed on the WEP/M-M sample, particularly at later stages. This occurs because corrosive factors degrade the MBI and PDA protective films over immersion time, resulting in their degradation and inability to offer long-term protection. Consequently, the WEP/M-M@P coating demonstrates superior long-term anticorrosion and self-healing properties.

**Figure 12:**
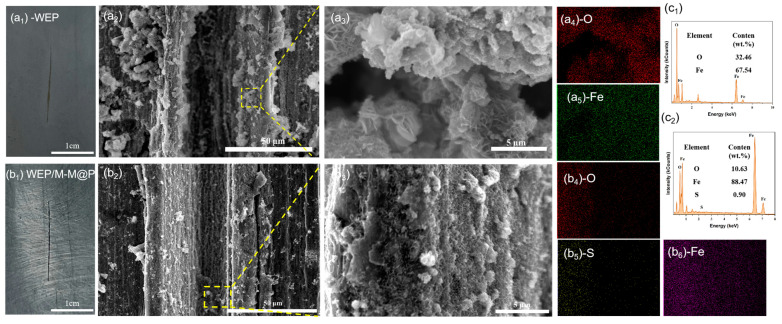
Optical images, surface morphology, and elemental composition of pure WEP and WEP/M-M@P coatings immersed in 3.5 wt.% NaCl solution at pH = 2 for 15 days: (**a_1_**) optical image of a pure WEP-coated sample, (**a_2_**,**a_3_**) SEM images of a pure WEP-coated sample, (**a_4_**,**a_5_**) corresponding element distribution map, (**c_1_**) corresponding EDS spectrum, (**b_1_**) optical image of a WEP/M-M@P-coated sample, (**b_2_**,**b_3_**) SEM images of a WEP/M-M@P-coated sample, (**b_4_**–**b_6_**) corresponding element distribution map, and (**c_2_**) corresponding EDS spectrum.

In addition, the corrosion morphology and elemental composition of the scratch coating were analyzed using SEM and EDS, and the results are shown in Figure 12. Figure 12a_1_–a_5_ clearly shows that a large number of corrosion products are evenly distributed in the scratch area of the WEP sample. The high-magnification SEM results show that these corrosion products present a loose, porous, dendritic micro-nano structure. EDS results show that these corrosion products are mainly iron oxides. From the above results, it can be concluded that at the defects of the pure WEP coating, the carbon steel substrate will undergo uniform corrosion and generate a large number of corrosion products. These products have a loose and porous structure and cannot effectively prevent further erosion by the corrosive medium. In the scratches of the M-M@P coating sample, more dense and stacked products can be seen. At the same time, the EDS results show that the S element is also detected in these products, indicating that MBI is released from M-M@P at the defects and participates in the corrosion reaction. The above results show that MBI plays a very important role in the corrosion inhibition process of carbon steel. It can participate in the corrosion reaction of carbon steel and generate a dense corrosion product layer covering the defects, thereby effectively inhibiting further corrosion of the carbon steel matrix.

**Figure 13:**
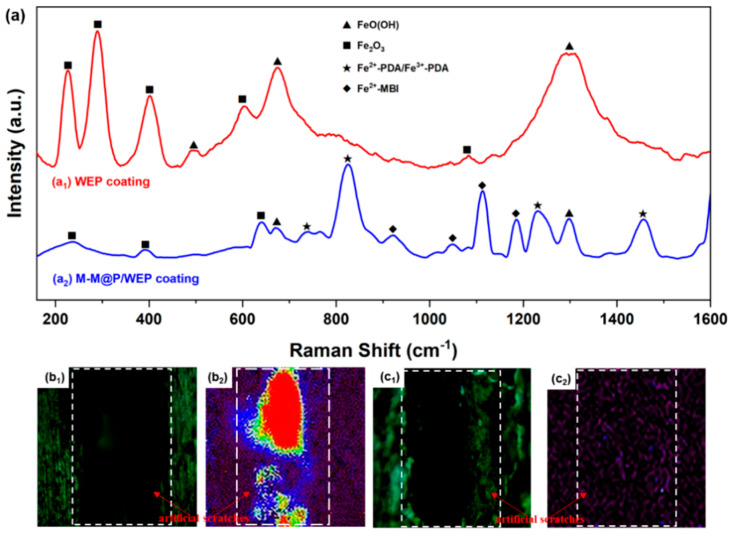
(**a**) Raman spectra of (**a_1_**) WEP- and (**a_2_**) WEP/M-M@P-coated specimens with artificial scratches after soaking for 20 days (after the coating is stripped off); (**b_1_**,**b_2_**) the Raman intensity spectra of pure WEP; (**c_1_**,**c_2_**) the Raman intensity spectra of WEP/M-M@P-coated specimen.

Microscopic laser Raman spectroscopy was utilized to examine the surface chemical composition of the scratched coating post-immersion, providing a detailed analysis of the self-healing mechanism of the WEP/M-M@P coating. Based on the results presented in Figure 13, the carbon steel sample with pure WEP presents a series of obvious Raman characteristic peaks of iron oxide corrosion products. Specifically, the peaks at 223, 243, 408, 608, and 1052 cm^–1^ in Figure 13a_1_ are assigned to α–Fe_2_O_3_ [61,62], while the peaks at 481, 665, and 1310 cm^–1^ are attributed to α–FeOOH and γ–FeOOH [61,63,64]. In contrast, the WEP/M-M@P-coated sample (Figure 13a_2_) presents a markedly different spectral profile. The peak at 734 cm^–1^ is assigned to the chelation between Fe ions and the catechol groups of PDA [65,66], and the peaks at 812, 1272, and 1483 cm^–1^ are the characteristic Raman vibration of dopamine [67]. Additionally, the peaks at 922, 1051, 1115, and 1187 cm^–1^ are assigned to the vibrational modes of MBI [68]. These observations confirm the formation of a PDA complex and a MBI corrosion inhibition film on the surface of the carbon steel substrate. Figure 13b_1_,b_2_,c_1_,c_2_ are the Raman intensity spectra of different substances on the sample surface. The surface of the WEP/M-M@P sample in Figure 13b_2_ has very strong Raman signals of Fe^2+^-MBI and Fe^2+^-PDA/Fe^3+^-PDA, which are distributed in a wide area. On the surface of the WEP sample, there is no signal of this product. Hence, a repair film is formed in the scratch area of the coating to inhibit the occurrence of corrosion reaction.

### 3.4. Self-Healing Mechanism of WEP/M-M@P Coatings

**Figure 14:**
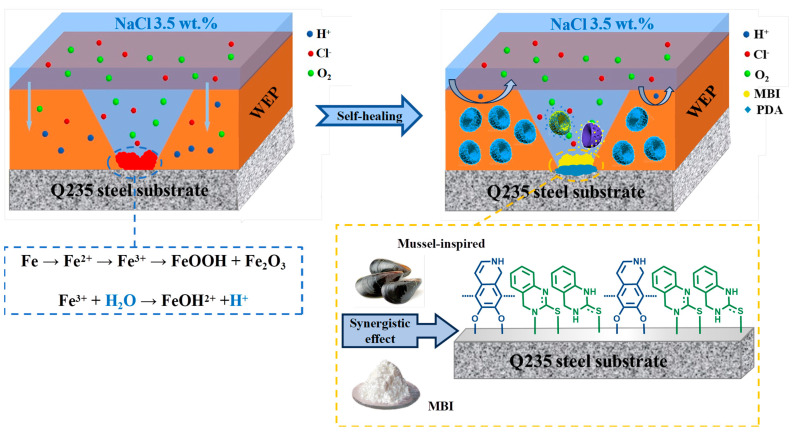
Self−healing mechanism diagram of a scratched WEP/M-M@P coating in an acidic medium.

Figure 14 presents a schematic representation of the self-healing protective mechanism of the M-M@P coating. During the preparation of the coating, microcracks and micropores are inevitably formed. These imperfections allow corrosive agents to infiltrate the coating matrix over time, leading to metal corrosion. The good dispersion of BMPDA in the epoxy coating sealed these pores, resulting in a significant enhancement in the penetration path of the corrosive electrolyte. On the other hand, the steel acts as an anode and loses electrons in electrochemical corrosion reactions, which results in a pH decrease through the accumulation of metal ions. The UV–vis absorption spectroscopy analysis confirmed that the acidic environment stimulated the release of MBI from the M-M@P. Therefore, the coating system containing corrosion inhibitors will quickly produce a dual protection function under the pH trigger caused by local corrosion. The BMPDA serves not only as a container for loading but also forms a chelate repair film with Fe^3+^, thereby acting as a corrosion inhibitor. This dual barrier effect of MBI and PDA effectively shields against corrosive media. In an acidic environment, the carbon steel substrate undergoes a hydrogen evolution corrosion reaction, and the reaction equation is as follows [45]:

(4) $$Fe\;\to {Fe}^{2+}\;+\;2e^{-}\;2H^{+}\;+\;2e^{-}\;\to \;2H_{2}$$

With the long-term penetration of electrolyte, the interaction between part of the substrate and the corrosive medium triggers the corrosion oxidize reaction at the interface [69]:

(5) $${Fe}^{2+}\;\to \;{Fe}^{3+}\;+\;e^{-}$$

The PDA forms a complex adsorption film on the surface of the steel substrate by anchoring Fe^2+^ and Fe^3+^ [70]:

(6) $${Fe}^{2+}+\mathrm{PDA}\to {Fe}^{2+}-\mathrm{PDA}\;{Fe}^{3+}+\mathrm{PDA}\to {Fe}^{3+}-\mathrm{PDA}$$

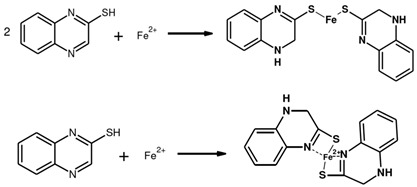
(7)

The MBI released from the scratches reacts with the Fe on the surface of the carbon steel substrate in two forms, including as a new MBI compound and as an MBI chelate, which is shown in the formula. This process forms a dual protective film on the surface of the substrate, effectively inhibiting corrosion reactions. The corrosion inhibitor promotes the formation of a passivation layer, thus hindering the further penetration of corrosive media into the dense protective film [69].

The above results show that the water-based epoxy coatings reinforced with M-M@P have significant anticorrosion properties and good self-healing properties compared with the pure water-based epoxy coatings.

## 4. Conclusions

In this study, the PDA-coated M-M@P anticorrosive nanofillers were prepared using emulsion-induced interfacial anisotropic assembly and a simple one-step synthesis method applicable to waterborne epoxy resin. The successful assembly of M-M@P nanomaterials was confirmed using TEM, SEM, FTIR, XPS, and HAADF-STEM. The MBI loading capacity of BMPDA was determined using thermogravimetric analysis, showing a loading capacity nearly 24.08%. The UV–vis absorption tests revealed significant variations in MBI release from M-M@P in the 3.5 wt.% NaCl solutions with different pH values, indicating acidic-responsive characteristics of the nanocontainers. Specifically, the release of MBI in the 3.5 wt.% NaCl solution at pH = 2 was 91.61%. Furthermore, the EIS and potentiodynamic polarization tests demonstrated that the M-M@P achieved a corrosion inhibition efficiency as high as 95.54% in the 3.5 wt.% NaCl solution at pH = 2. The addition of M-M@P significantly enhanced the protective performance of the scratched waterborne epoxy coatings, confirming the composite coating’s excellent corrosion inhibition performance. After 20 days of exposure to the 3.5 wt.% NaCl solution at pH = 2, the |Z|_0.01Hz_ for the WEP/M-M@P coating was three orders of magnitude higher than that of WEP coating alone. The M-M@P fillers demonstrated excellent anticorrosion and self-healing effects attributed to MBI release, which forms a passive repair film and reduces anodic corrosion activity on the surface of the carbon steel. In addition, the PDA can chelate with trivalent iron to form a protective film, further resisting corrosion reactions. Therefore, this study introduces a novel corrosion inhibitor loading system using BMPDA as the carrier and self-coated nanoparticles with PDA shells. This work provides a new design idea for developing smart nanocontainer-based protective coatings and exhibits significant potential in practical anticorrosion field. These coatings offer promising applications in acidic corrosive environments due to their outstanding anticorrosion and self-healing properties.

## Figures and Tables

**Table 1:** Electrochemical parameters of scratch coatings in 3.5 wt.% NaCl solution (pH = 2).

Samples	Time (d)	*R_P_* × 10^3^ (Ω·cm^2^)	*CPE_P_* × 10^−6^ (S s^−n^·cm^−2^)	n_p_	*R_L_* × 10^4^ (Ω·cm^2^)	*CPE_L_* × 10^−4^ (S s^−n^·cm^−2^)	n_L_	*R_ct_* × 10^4^ (Ω·cm^2^)	*CPE_d_* × 10^−6^ (S s^−n^·cm^−2^)	n_dl_
WEP	1	1.56	9.20	0.85	1.57	12.9	1	1.84	3.81	0.77
	3	1.21	24.7	0.82	1.04	0.06	0.69	1.18	1.27	0.98
	7	1.15	3.74	0.78	0.79	18.8	1	1.07	9.07	0.85
	15	1.49	60.9	1	0.51	0.52	0.86	0.99	2.72	0.73
	20	1.41	82.8	0.90	0.39	1.64	1	0.82	2.96	0.71
WEP/M-M	1	116.1	1.13	1	369	0.01	0.94	346.5	1.20	0.71
	3	174.4	1.04	0.80	107.5	0.09	0.74	107.4	0.001	0.93
	7	48.4	1.12	0.90	99.1	0.32	0.68	50.6	4.31	1
	15	89.2	2.67	0.61	20.1	0.02	1	23.9	4.04	0.51
	20	62.8	2.35	0.66	12.3	0.15	1	15.1	2.24	0.41
WEP/M-M@P	1	287.0	0.42	0.80	31.2	0.09	0.76	50.26	0.006	1
	3	394.7	0.89	0.95	384.4	0.07	0.76	651.6	1.24	0.80
	7	564.5	1.08	1	409.0	0.01	0.95	724.9	0.59	0.79
	15	359.6	0.68	0.85	435.4	0.06	0.79	751.4	0.097	0.76
	20	314.0	2.63	0.80	444.0	0.01	0.95	791.7	0.70	0.80

The standard deviation range for *R_P_* is between 1.3% and 6.6%. The standard deviation range for *CPE_P_* is between 4.3% and 8.1%. The standard deviation range for *R_L_* is between 2.8 and 4.9%. The standard deviation range for *CPE_L_* is between 0.7% and 7.1%. The standard deviation range for *CPE_d_* is between 4.9% and 9.3%. The standard deviation range for *R_ct_* is between 2.5% and 4.7%.

## Data Availability

The original contributions presented in this study are included in the article/Supplementary Materials. Further inquiries can be directed to the corresponding authors.

## Supplementary Materials

The following supporting information can be downloaded at: https://www.mdpi.com/article/10.3390/polym17162265/s1, Figure S1: Nyquist and Bode plots of carbon steel electrodes coated with different coatings immersed in the 3.5 wt.% NaCl solution at pH = 7 for different times: (a_1_), (a_2_), and (a_3_) WEP, (b_1_), (b_2_), and (b_3_) WEP/ M-M, and (c_1_), (c_2_), and (c_3_) WEP/ M-M@P; Figure S2: Nyquist and Bode plots of carbon steel electrodes coated with different coatings immersed in the 3.5 wt.% NaCl solution at pH = 4 after different times: (a_1_), (a_2_), and (a_3_) WEP, (b_1_), (b_2_), and (b_3_) WEP/M-M, and (c_1_), (c_2_), and (c_3_) WEP/M-M@P; Table S1: Electrochemical parameters of scratch coatings in 3.5 wt.% NaCl solution (pH = 7); Table S2: Electrochemical parameters of scratch coatings in 3.5 wt.% NaCl solution (pH = 4).
