# Supplementary material for: Acid-Responsive Self-Healing Waterborne Epoxy Coating: Preparation, Release Behavior, and Anticorrosion Performance Based on Bowl-Shaped Mesoporous Polydopamine Nanocontainer Loaded with 2-MBI Inhibitors
Source: Polymers (Basel). 2025 Aug 21;17(16):2265. doi: 10.3390/polym17162265 (PMC12389605; doi:10.3390/polym17162265)
Supplement: Supplementary file 1 [file polymers-17-02265-s001.zip › polymers-3814116-supplementary.pdf]

Supplementary Data

Acid-responsive self-healing waterborne epoxy coating: Preparation, release behavior and anticorrosion performance based on bowl-shaped mesoporous polydopamine nanocontainer loaded with 2-MBI inhibitors

Xiaohong Ji[b,c,1*], Minghui Yang[a,b,c,1], Huiwen Tian[b,c*], Jin Hou[a*], Jingqiang Su[d], Zhen Wang[d], Zixue Zhang[e], Yuefeng Tian[f], Liangliang Zhou[f], Guanghua Hu[e], Yunfei Yang[f], Jizhou Duan[b,c], Baorong Hou[b,c]

[a] Key Laboratory of Marine Chemistry Theory and Technology, Ministry of Education, College of Chemistry and Chemical Engineering, Ocean University of China, Qingdao 266100, China

[b] State Key Laboratory of Advanced Marine Materials, Key Laboratory of Marine Environmental Corrosion and Bio-fouling, Institute of Oceanology, Chinese Academy of Sciences, Qingdao, 266071, China

[c] Center for Ocean Mega-Science, Chinese Academy of Science, Qingdao 266071, China

[d] Shandong Railway Investment Holding Group CO., LTD, Jinan, 250014, China

[e] Jiqing High Speed Railway Co., Ltd., Jinan, 250014, China

[f] Lunan High Speed Railway Co., Ltd., Jinan, 250000, China

*Corresponding authors: Xiaohong Ji (E-mail: jixiaohong@qdio.ac.cn)

Huiwen Tian (E-mail: tianhuiwen@qdio.ac.cn)

Jin Hou (E-mail:) (E-mail: houjinqd@163.com)

As depicted in Fig. S1(a_2), the impedance modulus value (|Z|) of the WEP coating at a frequency of 0.01 Hz after 20 days of immersion decreased to 1.67×10^4 Ω·cm². This decline suggests that the corrosive medium gradually penetrated the coating's scratches during immersion time, leading to a reduction in the impedance modulus of the WEP coating. Consequently, the WEP coating was unable to provide adequate corrosion protection. Based on Fig. S1(b_2), the WEP/M-M coating showed the highest

|Z| value of 1.34×10^5 $\Omega \cdot cm^2$ after immersion for 15 days. The corrosion inhibitor of the M-M-doped coating is released more slowly in the 3.5 wt.% NaCl solution with pH = 7. The decrease in $|Z|_{0.01Hz}$ after 15 days may be due to the depletion of MBI. It was proved that in neutral solution, the MBI from the M-M was not completely released. In the case of M-M@P coating, as shown in Fig. S1(c_2), after immersion in $|Z|_{0.01Hz}$ for 10 days However, the impedance value of the WEP/M-M@P coating rebounded on the 15th day, indicating that the penetration of the NaCl solution destroyed the integrity of the coating. Subsequently, the corrosion inhibitor was released from the M-M@P nanocontainer and adsorbed on the surface of the steel substrate, preventing the electrolyte from contacting the steel substrate again. This self-healing effect, based on the on-demand release of MBI from the nanocontainers to provide durable protection on the scratched area, was not observed for the other studied coatings. Fig. 8(a) shows the $|Z|_{0.01Hz}$ after immersion for 20 days. It can be seen that the corrosion resistance and self-healing properties of the M-M@P coating are higher than the other coatings.

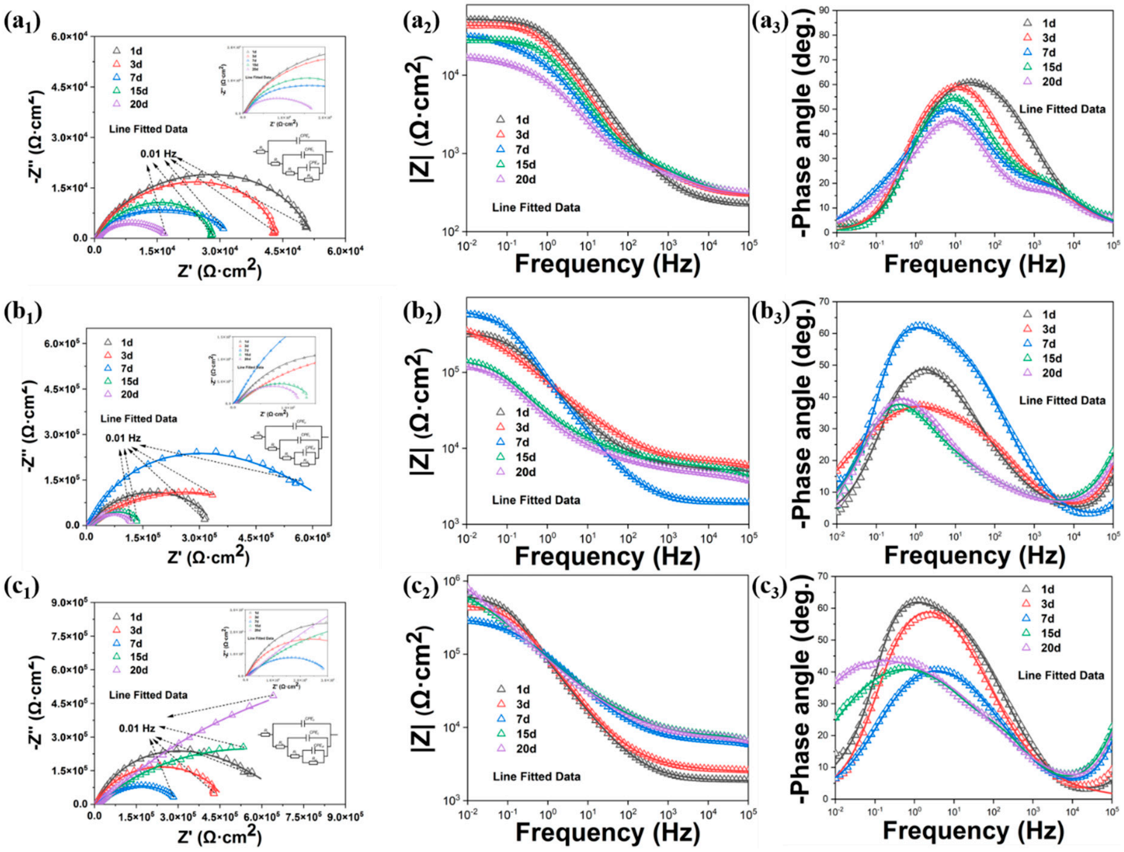

Fig. S1. Nyquist and Bode plots of carbon steel electrodes coated with different coatings immersed in the 3.5 wt.% NaCl solution at pH = 7 for different times: (a_1), (a_2), and (a_3) WEP, (b_1), (b_2), and (b_3) WEP/ M-M, and (c_1), (c_2), and (c_3) WEP/ M-M@P.

Fig. S2 illustrates the EIS results for various scratched coating samples submerged in a 3.5 wt.% NaCl solution at pH 4 over a period of 20 days. As depicted in Fig. S2(a_1-a_3), the impedance arc radius of the uncoated sample significantly diminishes with time. Additionally, the magnitude of the impedance, |Z|, decreases from 6.86×10^4 Ω·cm² on the first day to 1.54×10^4 Ω·cm² by the 20th day. When the $|Z|_{0.01Hz}$ is plotted against time, a decreasing trend of |Z| can be observed (Fig. 8(b)), indicating that the blank coating has no self-healing ability. The electrochemical parameters derived from fitting

the equivalent circuit model are detailed in Table S2. These parameters provide deeper insights into the corrosion characteristics of the metal substrate in the scratched regions. It can be seen from Table S2 that the value of the charge transfer resistance R_{ct} decreases with the increase of time. The R_{ct} represents the corrosion reaction rate in the metal interface area. The smaller the R_{ct} value, the faster the corrosion reaction rate. Therefore, the blank coating with scratches is severely corroded after a while, and the coating has failed. The radius of the capacitance loop of WEP/M-M coating gradually decreases by prolonging the immersion time. This result indicates that the corrosion inhibitor may be depleted due to its direct release. In addition, the MBI membrane cannot prevent corrosion for a long time, and it can only delay the corrosion process. Therefore, the |Z| value decreases with time. This trend can be seen from the $|Z|_{0.01Hz}$ in Fig. 8(b). This result indicates that the WEP/M-M coating quickly releases the MBI corrosion inhibitor on the carbon steel surface, and the formed passive film plays a shielding role against the corrosion reaction. After immersion, the corrosion factors erode the MBI protective film. Therefore, the |Z| value decreases slightly and gradually reaches a stable value as shown in Nyquist plots (Fig. S2(c_1)). The impedance arc radius of the EIS test on the first day is smaller due to the scratches in the coating, and the exposed carbon steel substrate undergoes anodic dissolution when exposed to the corrosive solution. However, the impedance arc radius increases after 7 days of immersion as the immersion time increases. This occurrence is attributed to the development of a protective MBI film in the scratched regions, facilitated by the release of the corrosion inhibitor from M-M@P. Figure S2(c_2) reveals that on the 7th day, the impedance of the

WEP/M-M@P coating increased, signifying that the coating exhibits effective anti-corrosion and self-healing capabilities.

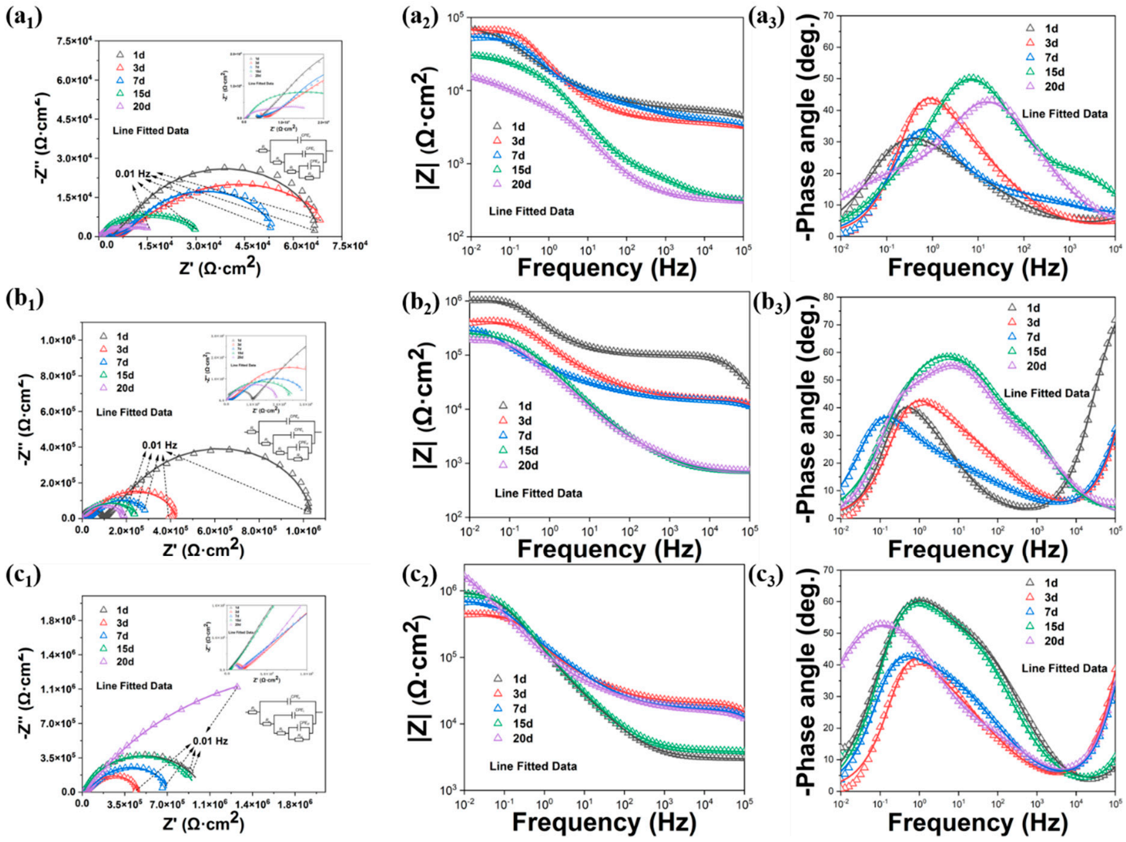

Fig. S2. Nyquist and Bode plots of carbon steel electrodes coated with different coatings immersed in the 3.5 wt.% NaCl solution at pH = 4 after different times: (a_1), (a_2), and (a_3) WEP, (b_1), (b_2), and (b_3) WEP/M-M, and (c_1), (c_2), and (c_3) WEP/M-M@P

Table S1 Electrochemical parameters of scratch coatings in 3.5 wt.% NaCl solution (pH = 7)

Samples	time (d)	R_s (Ω·c	R_P $\times10^4$ (Ω·cm^2)	CPE_P $\times10^{-6}$ (S s^{-n}·c	n_p	R_L $\times10^3$ (Ω·cm^2)	CPE_L $\times$ 10^{-4} (S	n_L	R_{ct} $\times10^3$ (Ω·cm^2)	CPE_d $\times10^{-5}$ (S s^{-n}·	n_{dl}

		m ^{2})		m^{-2})			s^{-n}·c m^{-2})			cm^{-2})	
WEP	1	-	3.15	6.15	0.76	3.12	5.26	0.32	3.12	1.38	0.95
	3	-	2.24	8.48	0.78	0.40	0.15	0.58	20.5	1.54	0.99
	7	-	0.67	22.0	0.88	26.2	0.35	0.64	0.44	1.09	0.61
	15	-	1.66	10.1	0.79	10.8	0.24	0.57	0.64	1.49	0.57
	20	-	0.69	27.7	0.77	0.36	0.09	0.64	10.1	8.63	0.66
WEP/M-M	1	-	4.43	11.0	0.99	30.2	0.03	0.77	31.3	1.67	0.42
	3	-	6.38	6.56	0.85	35.0	0.08	0.66	39.7	5.11	0.53
	7	-	8.72	7.42	1	39.2	0.04	0.98	51.6	5.96	0.62
	15	-	5.15	4.65	0.75	10.6	0.25	0.80	12.8	1.63	0.76
	20	-	10.80	16.3	0.75	4.08	0.46	0.75	10.4	1.33	0.33
WEP/M-M@P	1	-	1.85	0.75	1	66.9	0.06	0.62	95.4	27.0	0.98
	3	-	2.34	35.6	0.41	35.0	0.03	0.80	48.4	28.3	0.81
	7	-	9.91	6.89	0.51	21.1	0.05	0.68	26.2	37.1	0.89
	15	-	7.67	11.4	0.82	57.9	0.04	0.55	69.4	20.9	0.74
	20	-	5.86	17.9	0.97	67.4	0.05	0.38	99.4	17.19	0.61

Table S2 Electrochemical parameters of scratch coatings in 3.5 wt.% NaCl solution (pH = 4)

Samples	time (d)	R_s (Ω·cm^{2})	R_P ×10^{4} (Ω·cm^{2})	CPE_P ×10^{-5} (S s^{-n}·cm^{-2})	n_p	R_L ×10^{4} (Ω·cm^{2})	CPE_L ×10^{-4} (S s^{-n}·cm^{-2})	n_L	R_{ct} ×10^{4} (Ω·cm^{2})	CPE_d ×10^{-6} (S s^{-n}·cm^{-2})	n_{dl}
WEP	1	-	0.54	0.90	0.70	4.91	0.32	0.43	6.98	36.70	0.77
	3	-	1.62	2.13	0.54	3.24	0.13	0.96	4.66	0.021	0.62
	7	-	3.93	1.85	0.87	2.38	0.008	0.87	3.76	23.34	0.35

	15	-	2.47	3.56	0.64	0.45	0.11	0.61	3.48	21.58	0.87
	20	-	1.66	12.5	0.51	0.32	0.078	0.29	1.37	30.50	0.77
WEP/M-M	1	-	28.66	0.14	0.61	99.5	0.014	1	115.1	0.41	0.98
	3	-	1.45	0.07	0.94	36.9	0.030	0.51	49.70	2.17	0.91
	7	-	1.35	0.22	0.93	26.95	0.16	0.89	30.93	9.20	0.40
	15	-	26.28	0.40	0.74	12.90	0.042	0.72	21.00	0.14	1
	20	-	0.13	0.66	0.63	4.61	0.058	0.73	9.60	7.76	0.92
WEP/M-M@P	1	-	1.89	0.49	0.60	72.95	0.006	1	98.03	1.81	0.82
	3	-	11.8	0.31	0.50	32.55	0.022	0.92	48.00	0.65	0.95
	7	-	2.79	0.27	0.52	46.14	0.002	0.91	69.96	3.24	0.91
	15	-	0.37	0.07	1	77.49	0.048	0.59	90.55	1.76	0.86
	20	-	1.52	0.02	0.92	251.6	0.030	0.52	409.3	3.05	0.69
